# Neonatal onset of bipolar spectrum disorder through a three-generation familial study

**DOI:** 10.1192/j.eurpsy.2022.1034

**Published:** 2022-09-01

**Authors:** B. Abdelmoula, S. Sellami, M. Keskes, N. Bouayed Abdelmoula

**Affiliations:** Medical University of Sfax, Genomics Of Signalopathies At The Service Of Medicine, Sfax, Tunisia

**Keywords:** Bipolar spectrum disorder, heritable mood/mental disorder, Longitudinal familial study, Neonatal onset

## Abstract

**Introduction:**

Age at onset of pediatric bipolar spectrum disorder (BSD) is an important marker of a more severe form and a highly heritable mood/mental disorder.

**Objectives:**

Here, we report a familial Tunisian BSD follow-up study showing a very early onset of the BSD at the neonatal period.

**Methods:**

A 28-year-old female and her 30-year old sister were referred for genetic and psychological assessments due to recurrent depressive episodes.

**Results:**

Psychological assessment revealed a BSD type II with episodes of hypomania for both patients. The 30-year old sister presented a mixed form of BSD coupled with autistic traits, hyposomnia and obsessive-compulsive behaviors. Intellectual and cognitive abilities were without concerns. Familial history revealed BDS among paternal relatives including the brothers’ and sisters’ father as well as all their uncles offspring’s, and their grandparents, who were consanguineous. The depressive mood was a common sign in the three generations. Personal history revealed significant signs of a very early onset of the disorder since the neonatal period for the two sisters as well as for their four paternal cousins who also presented BSD features. Familial risk of BSD in this family correlates with a variably higher personal risk of other psychiatric disorders such as anxiety, drug abuse, personality disorders, and autism spectrum disorder.

**Conclusions:**

Environmental conditions, familial care and educational level have a strong correlation with the severity and the efficiency of cognitive management of BSD and its psychiatric comorbidities. BSD is highly heterogeneous and polygenic and personalized management has considerable clinical repercussions benefits.

**Disclosure:**

No significant relationships.

